# Family involvement in geriatric hospitalization: a qualitative analysis of state regulations in Israel

**DOI:** 10.1186/s13584-025-00709-0

**Published:** 2025-07-23

**Authors:** Keren Semyonov-Tal, Eldad Davidov

**Affiliations:** 1 Institute of Sociology and Social Psychology, University of Cologne, Cologne, Germany; 2 Department of Labor Studies, Tel Aviv University, Tel-Aviv, Israel

**Keywords:** Responsiveness, Social networks, Family participation, Geriatrics, Qualitative methods

## Abstract

**Background:**

The growing recognition of the importance of family involvement in geriatric healthcare underscores the need to examine how regulatory frameworks, such as health regulations, shape family involvement in hospital care. The study’s objectives are twofold. First, it aims to examine how health regulations shape family involvement in geriatric care. Second, the study highlights areas where policies can be adjusted to foster greater family participation.

**Methods:**

We conducted a qualitative analysis of over 150 regulations related to geriatric care in Israel, focusing on how these regulations facilitate family participation as an organizational goal that could either support or hinder family involvement in the care process.

**Results:**

Our findings reveal that various government documents largely promote and encourage family involvement to foster a more responsive and effective healthcare system. We identified several key aspects of family participation in geriatric treatment, including information sharing and communication, care planning and decision-making, advocacy, and emotional and social support. This study makes two primary contributions. First, it provides a detailed examination of how regulations strengthen the role of families in the geriatric healthcare environment, thereby reducing organizational overload and enhancing patients’ well-being. Second, it highlights the need for legal reform to better incorporate and integrate family involvement in geriatric care, addressing contradictions between real-world practices and regulatory frameworks to meet the evolving needs of the older population.

**Conclusions:**

The study offers insights for policymakers and healthcare providers seeking to develop regulatory frameworks that enhance family involvement in geriatric healthcare.

## Background

Families play a crucial role in planning and evaluating care in health settings, significantly influencing both the quality of care and the well-being of patients [1, 2]. In geriatric hospital settings, family involvement can include providing emotional support, actively participating in care planning, and advocating for the needs and preferences of older loved ones [3]. The study contributes to the fields of responsiveness and social support networks by examining how regulatory frameworks, particularly health regulations, shape and promote family involvement in geriatric hospital care (i.e., long-term geriatric departments and active geriatric departments that provide complex nursing, rehabilitative care, and subacute geriatric services). Despite the widely acknowledged importance of family engagement, only limited research has explored how family participation is reflected in formalized regulations. The study’s objectives are twofold. First, it aims to examine how regulatory frameworks, specifically health regulations, shape family involvement in geriatric care. Second, the study seeks to highlight areas where policies can be adjusted to foster greater family participation, ultimately enhancing the quality of geriatric care.

### Responsiveness

Responsiveness refers to how patients are treated during their interactions with healthcare providers and systems and throughout their hospital stays [4–8]. The World Health Organization (WHO) emphasizes health system responsiveness as a fundamental organizational goal [1]. According to the WHO, responsiveness consists of seven key components: respect for patient dignity, autonomy, confidentiality, prompt attention to health needs, basic amenities, choice, and social support networks [1]. Among these components, social support networks, including family visits and support, are crucial for responsive care, improve health outcomes, and are a valued aspect of the caregiving experience [1, 2].

### Family participation

Family involvement can significantly influence the quality of care and the well-being of patients [2]. Families provide crucial emotional support, which is essential for the recovery of elderly individuals [9]. Family members actively participate in care plan meetings and decision-making, contributing to discussions about health impacts and medical conditions [10]. A comprehensive literature review by Wei et al. [3] identified four core elements of family involvement in care: (1) maintaining contact with the patient, (2) engaging in care activities, (3) planning and monitoring care, and (4) providing support to the patient. Family members are increasingly encouraged to participate in their loved one’s medical care, thereby advocating for the needs and preferences of older patients and ensuring that their voices are heard [3]. Puurveen et al. [11] found that family members adopt diverse roles in geriatric care, which can be categorized into four primary areas. First, they provide hands-on assistance with personal care tasks (which are typically staff responsibilities) such as bathing and grooming. Second, they oversee and manage care by facilitating appointments and advocating for their relative’s rights. Third, they offer emotional support by maintaining routines, organizing leisure activities, and serving as a connection to the outside world. Fourth, they contribute to the broader community by engaging in social activities and assisting others [11].

Involving families in patient care provides nursing staff with valuable insights into the things that matter most in a patient’s life [12]. For example, family participation plays a significant role in facilitating future planning conversations [13]. However, while family involvement can enhance the incorporation of family viewpoints in care planning, participation often varies among family members and may be limited [14]. Decision-making for hospitalized elderly patients, especially for those facing life-threatening situations, is significantly influenced by family dynamics. Families often carry emotional burdens as they consider the patient’s prognosis and reflect on the patient’s presumed wishes. This deliberation can result in considerable psychological stress [15]. Additionally, the nature of the family–patient relationship plays a crucial role in decision-making, with closer relationships typically resulting in more active participation in care decisions [15].

### The Israeli context

Israel has a comprehensive healthcare system, encompassing a vast network of clinics, public hospitals, and preventive healthcare services [16]. The government primarily finances the healthcare system and guarantees universal access to national health insurance for all permanent residents, regardless of gender, ethnicity, or background [17]. Despite occasional budgetary constraints and prioritization of specific treatments [18], public healthcare funding covers most patient needs. The Patient’s Rights Act of 1996 (PRA) [19] mandates that all residents are entitled to receive respectful and dignified care during hospitalization while safeguarding their legal rights [20]. This includes specific provisions addressing the medical needs of geriatric patients [21]. Geriatric hospitals in Israel are specifically designed for individuals aged 65 and older who experience age-related issues such as memory loss and dementia. These hospitals are governed by the People’s Health Ordinance of 1940 [22] and the People’s Health Regulations (Registration of Hospitals) of 1966 [23], which mandate that all medical facilities, including geriatric hospitals, must obtain approval from the Ministry of Health prior to establishment or operation. According to the procedures set for a geriatric hospital (Procedure 0.2.1), a geriatric hospital is defined as “a hospital for long-term care that includes one or more departments for nursing or mentally ill patients, which meets the necessary licensing requirements and operates within the framework of the license granted by the Ministry of Health. The hospital may also include departments for complex nursing care”.

### The legal framework

Despite the crucial role that families play in geriatric care, Israeli law provides only limited recognition of their involvement in medical decision-making. The PRA [19] serves as a framework for ensuring patient autonomy, particularly emphasizing the necessity of informed consent for medical treatment from patients capable of making their own medical decisions. However, it only offers limited provisions for integrating family members into decision-making. Specifically, Sect. 13 of the PRA [19] mandates that no medical treatment can be administered without the patient’s explicit consent. While this provision highlights the importance of patient autonomy, it also restricts the ability of family members to participate in medical decisions—even though their support and insights could provide valuable context regarding the patient’s preferences and values. However, Sect. 16 of the PRA offers a small degree of family involvement by permitting patients to appoint a proxy—a chosen individual who can make medical decisions on their behalf if they become incapacitated. However, this still reflects a narrow understanding of family dynamics, especially in geriatric hospitalization, as it reduces the family’s role to that of passive bystanders unless designated explicitly as proxies by the patient. Complicating matters, Sect. 15(2) of the PRA [19] grants healthcare providers the authority to administer treatment even against a patient’s expressed wishes in life-threatening situations. In such cases, the input of family members is not considered, highlighting the vast disconnect between the emotional and social support that families can offer [9] and the rigid legal structures in place. Section 6A of the PRA permits the presence of family members during treatment to provide emotional support to the patient but this provision does not extend to granting them any formal role in decision-making.

The Terminally Ill Patient Law of 2005 [24] further complicates matters by imposing limitations on the rights of family members in cases involving competent adults facing end-of-life decisions. The law assumes a preference for life-prolonging treatments unless the patient has explicitly stated otherwise, reflecting a lack of trust in the family’s ability to represent the patient’s wishes accurately. Nevertheless, if the patient’s preferences are unknown and an urgent medical decision is required, the law requires physicians to consult the patient’s relatives to assess their perspective on life-sustaining treatments (Sects. 5 and 14 of the Terminally Ill Patient Law).

The Legal Capacity and Guardianship Law of 1962 [25] reflects a strong dedication to upholding an individual’s right to make choices about their personal matters and honoring their autonomy. A vital part of this framework is the Durable Power of Attorney, introduced in the 2016 amendments to the Guardianship Law [25] (specifically, Amendment 18). This legal tool enables individuals to designate decision-makers while they are still mentally competent, allowing them to specify the range of decision-making powers given, encompassing medical, financial, and personal care choices. Consequently, patients can establish a durable power of attorney that outlines the decision-making responsibilities of their family. This setup honors individual autonomy while recognizing that not every patient wants to involve others in their healthcare decisions; some might feel that family dynamics could complicate their treatment or may prefer to make healthcare choices on their own.

The most recent development in this legal landscape is the introduction of the Procedure regarding the appointment of a decision supporter by the court in 2018 [26] and the Legal Capacity and Guardianship (Supported Decision-Making) regulations in 2024 [27]. These regulations introduce a mechanism through which the court can appoint a temporary decision-making supporter for an adult who, with assistance, can make decisions regarding their affairs. This innovative approach addresses the limitations of previous decision-making mechanisms by providing a more flexible option for individuals. The decision supporter, who can be a family member, does not make decisions on behalf of the individual they are assisting. Instead, the supporter can only help facilitate the decision-making process, prioritizing individual autonomy while acknowledging the practical challenges of geriatric care.

Therefore, the study aims to explore how regulatory frameworks shape and promote family involvement in geriatric hospital care and highlight areas where policies can be modified to enhance greater family participation.

## Methods

In 2007, the Ministry of Health published an updated set of procedures for geriatric hospitals, which includes over 150 formal regulations for medical personnel (i.e., The Geriatric Hospital Procedures Manual, Ministry of Health– Geriatrics Division) [28]. According to the Ministry of Health website, the Geriatric Hospital set of procedures forms the basis for the work of the entire geriatric system, both in long-term geriatric departments (wards for nursing and mentally ill patients) and in active geriatric departments such as complex nursing, prolonged ventilation, rehabilitative geriatrics, and subacute geriatrics [29]. The documents contain detailed regulations on quality control, safety, and provider-patient relationships. As part of a broader study on geriatric hospitals in Israel, we conducted an in-depth analysis of these 150 documents, comprising 94 procedural guidelines for geriatric hospital management for effectively running a geriatric hospital and 56 circulars and procedural updates issued between 2009 and the present. For this study, we focused specifically on different aspects of family participation to address our research question.

We employed a qualitative thematic analysis approach to examine the written information [30, 31]. Data analysis was conducted using both manual and software-based AI (atlasti.com) methods to analyze the data, identifying recurring codes and connections. The data obtained from this process were organized into preliminary subthemes, which were reviewed in collaboration with expert colleagues to ensure a consistent and logical arrangement of themes and subthemes. Relevant quotes were translated from Hebrew to English, and findings were documented by presenting themes, relevant notes, and data excerpts to ensure credibility and transferability.

Our coding process followed a spiral approach [32]. Initially, we analyzed the data using classic thematic analysis without specifically considering the literature. As specific themes began to emerge, we revisited the literature to refine our findings based on the interaction between the literature and our analysis results. The study was based on publicly available data.

## Results

In the following section, we provide a detailed description of the categories reflected in the data. Our analysis indicates that family involvement in geriatric treatment in Israel is strongly encouraged, as evidenced by the frequent appearance of the word family.^1^ Table 1 outlines the major recurring themes and subthemes related to family support and participation in geriatric hospitals.

**Table 1:** Themes and subthemes for family participation in geriatric hospitals

Theme	Subtheme	Description
Information Sharing and Communication	Initial Information Gathering	Comprehensive data collection from the family
	Ongoing Communication	Regular, proactive updates about the patient’s condition
Care Planning and Decision Making	Collaborative Treatment Planning	Active involvement of family in developing care plans
	Informed Consent	Obtaining family consent for medical procedures
Advocacy	Patient Rights	Protecting patient rights
	Privacy and Intimate Care Considerations	Advocating for patient preferences in personal care
Emotional and Social Support	Continuous Engagement	Encouraging ongoing family connections with the patient
	Support for Families	Supporting families during critical medical and challenging situations
	Adaptation Support	Helping patients adjust to new living arrangements

### Information sharing and communication

Formalized guidelines recognize the value of family involvement in the initial assessment, during the adaptation of geriatric patients to their new living arrangements, and when assessing their medical situation. Families are encouraged to actively contribute to the data collection process:

*“Collecting information from the family—During the diagnosis and assessment*, *the nurse will emphasize comprehensive collection of data from the family” (Procedure 2.2.2: Initial intake of a mentally frail patient).*

This data collection (from family) will include: *“[…] the patient’s personal*, *family*, *and medical history as well as the patient’s previous habits*, *including those related to functioning and lifestyle.” (Procedure 2.2.2: Initial intake of a mentally frail patient)*. This process ensures that the preferences, needs, and expectations of both patients and their families are acknowledged and addressed.

Family participation is not seen as a one-time event but rather as an ongoing process:

“Creating quality communication between the nurse and the patient and his/her family contributes to building reciprocal relationships.” (Procedure 2.3.6: Promoting communication between the nurse and the patient and family).

This includes providing proactive updates about the patient’s condition, encouraging dialogue, and ensuring transparent communication among patients, families, and medical staff:

*“The social worker will inform the patient’s family of the conclusions of the team meeting” (Procedure 4.0.4: Integration of the social worker into the multidisciplinary team).*

### Care planning and decision making

Formalized guidelines emphasize the importance of involving families in developing care and treatment strategies, as well in risk prevention planning and discussions. This approach establishes family members as active partners in the care planning process:

*“The purpose of the procedure is to determine an intervention plan in collaboration with the multidisciplinary team*, *family members*, *and others significant to the patient.” (Procedure 2.2.2: Initial intake of a mentally frail patient)*.

An important organizational goal is to ensure that families are involved in critical medical decisions, including obtaining consent for medical procedures, and representing patients who are unable to make decisions independently:

*“The entire process will be carried out according to the principles of supportive care*, *with a view to the patient’s comfort and dignity. At each stage*, *it is important to inform the patient and the family and obtain the patient’s consent or the consent of the patient’s proxy/guardian.” (Circular 41/2014: Enteral nutrition for adults)*.

### Advocacy

Family advocacy mechanisms were highlighted in many documents. This includes providing families with the tools to advocate for and exercise patient rights:

*“The geriatric hospital management will appoint someone responsible for protecting the patient’s rights*, *whose roles will […] include […] Receiving complaints from patients and their families; Counseling and assisting patients and their families in all matters related to exercising their rights.” (Procedure 0.1.2: Patient rights)*.

Advocating for patient preferences includes, for example, providing gender-sensitive care options and managing family expectations and presence during sensitive procedures:

*“The elderly patient’s and/or family’s bathing and dressing preferences should be checked: frequency*, *timing during the day (morning*, *evening)*, *expectations that a woman or man will provide assistance*, *sensitivity to substances*, *types of bathing*, *bathing time.” (Procedure 2.3.10: Maintaining the patient’s personal hygiene)*.

### Emotional and social support

Encouraging continuous family connections, facilitating regular visits, and integrating families into hospital activities and events were identified in the documents as key organizational goals, emphasizing the role of families in providing emotional support to patients:

*“The nurse will encourage maintaining contact with the patient’s family and others significant to the patient upon admission*, *during hospitalization*, *and towards discharge.” (Procedure 2.1.1: Principles of nursing practice)*.

Emotional and social support includes not only families aiding patients but also medical staff providing support to families during critical medical moments. This support encompasses bereavement assistance and emotional care in challenging situations:

*“The death of a patient constitutes a stressful situation for the family*, *the care team*, *and the other patients. Treatment of this condition requires action to maintain the dignity of the deceased and support the family and staff.” (Procedure 0.5.6: Death and care of the deceased and the family)*.

Families are also encouraged to assist geriatric patients by helping them adapt to new living environments and maintain emotional connections, and by facilitating visits and interactions:

*“To facilitate the transition and change in the life of the elderly*, *it is important to act professionally and empathetically. The patient’s transition process will be carried out with the participation of the family and the entire multidisciplinary team.” (Procedure 0.5.1: Receiving a patient)*.

## Discussion

The study’s first goal was to examine how formal regulations promote and shape family involvement in geriatric hospitalization. The analysis identified several key aspects of family participation in geriatric treatment, including information sharing and communication, care planning and decision-making, advocacy, and emotional and social support. These themes illuminate the multifaceted role of families in enhancing care quality.

The first theme, information sharing and communication, is fundamental to family involvement in geriatric healthcare. This begins with gathering initial information, which helps establish a comprehensive understanding of the patient’s medical history and needs, fostering trust between the healthcare team and the family. Ongoing communication strengthens this relationship by allowing all parties to provide regular updates on the patient’s condition. This comprehensive communication strategy can empower families to feel more connected and informed during hospitalization [15].

The second theme focuses on family participation in care planning and decision making and plays an influential role in shaping medical outcomes. Collaborative treatment planning allows families to voice their concerns and preferences, leading to care plans that are both medically sound and aligned with the patient’s values and wishes [10]. Obtaining consent from family members for medical procedures underscores the ethical obligation of involving them in decisions that affect their loved one’s life [15]. This process also reinforces the family’s role as patient advocates who help safeguard their rights and preferences throughout the care process.

Advocacy emerges as a third key theme, reflecting the family’s critical role in protecting patient rights and preferences. Families advocating for specific patient needs, such as privacy and intimate care considerations, create a more respectful and personalized care environment. Family advocacy allows relatives to alert healthcare providers to potential patient dignity and autonomy concerns, ensuring that the patient’s voice is prioritized and heard [3]. This advocacy also helps ensure that the healthcare system remains responsive to the challenges faced by older patients [5, 6], whose needs often differ from those of younger populations.

Emotional and social support of family members and patients emerges as a fourth theme and plays a crucial role during hospitalization [9]. Continuous family engagement and support fosters strong patient–family member relationships, which can lead to improved emotional well-being and a faster recovery. Moreover, supporting families during critical medical situations benefits not only the patient but also helps family members cope with stress by providing them with the necessary resources to navigate difficult times [11]. The emerging subtheme of adaptation also indicates the importance of helping patients adjust to changes in their living arrangements, particularly following hospitalization [3].

In sum, the study highlights the critical role of family involvement in geriatric hospitalization as emphasized by formal regulations. These regulations promote open communication between families and healthcare providers, ensuring that families are informed and engaged in their loved one’s care. They encourage family participation in care planning and decision-making, reinforcing the ethical obligation to align medical care with the patient’s preferences and will. The regulations recognize families as advocates for patients, enabling them to protect the patient’s rights and preferences. Furthermore, by acknowledging the emotional and social needs of both patients and their families, these regulations facilitate a supportive environment that contributes to recovery and helps families cope with the stresses of hospitalization.

The study’s second goal was to identify areas where policies and legislation can be adjusted to foster greater family participation. Despite emphasizing family involvement in official healthcare policies, as codified in the regulations, Israeli law prioritizes patient autonomy over family involvement [see Table 2]. These legal frameworks often delegate family members to the role of passive or partial observers, limiting their participation in involvement and decision-making, particularly in geriatric medicine, where patients may struggle to communicate their wishes. For example, the Guardianship Law [25] permits patients to create a durable power of attorney, which outlines the family’s role in decision-making or allows for the appointment of a decision supporter. However, these documents must be completed while the patient is legally competent. In practice, many patients do not prepare such documents in advance. Another instance in which Israeli law specifically addresses family participation relates to financial needs. For example, Israeli law establishes a legal obligation for adult children to provide for the basic needs of their elderly parents. This obligation stems from the Child Support Enforcement Law of 1979 [33] and Guardianship Law of 1962 [25], which mandates that children provide financial support to their parents during times of distress. However, aside from these, and to the best of our knowledge, no other institutional mechanisms are currently in place to require or track family participation in geriatric treatment.

**Table 2:** Legal tools (Pros and Cons)

Legal Tool	Pros	Cons
Patient Rights Act (PRA) [19]	Ensures patient autonomy Allows proxy appointment Permits family presence for emotional support Protects from unwanted interventions Clear consent framework	Restricts family participation Family as passive bystanders unless proxies No formal decision-making role Overlooks family context Rigid legal structure
Terminally Ill Patient Law (2005) [24]	Clear end-of-life protocols Physician-family consultation in urgent cases Structure for emergency decisions Legal certainty in sensitive situations Family input when preferences are unknown	Limits family rights Assumes life-prolonging preference May override family insights
Guardianship Law (1962 and 2016 Amendments) [25]	Supports individual autonomy Enables formal family decision-making Customizable decision powers Honors patient choice	Requires advance planning May not address unexpected situations Requires formal arrangements
Legal Capacity and Guardianship (Supported Decision-Making) (2018, 2024) [26, 27]	A temporary supporter can be appointed Balances autonomy and assistance More flexible framework	Supporters cannot make decisions Requires court involvement

These legal frameworks highlight a significant gap between legal theory and regulations, emphasizing the need to reevaluate how family roles are supported and formalized in medical processes. While Israeli geriatric hospitals acknowledge the importance of both patient autonomy and family support, the current legal framework mostly restricts family influence in critical decisions, marginalizing individuals who are vital in providing care.

## Future policies

The study’s findings highlight the need for legal reforms to integrate family involvement in geriatric care better. As presented in the results and discussion, the current discrepancy between healthcare policies and legal frameworks highlights the necessity of aligning legislation with the realities of patient care and family dynamics. As healthcare becomes increasingly complex and personalized, balancing personal autonomy with family involvement in medical decision-making has emerged as a significant issue for legal debate [34]. For example, Taiwan has encountered challenges in balancing patient autonomy and family input in terminal illness cases [35], while U.S. models highlight how communal decision-making can enhance patient autonomy [36]. Therefore, the legislator must acknowledge the essential role of family involvement during critical times. Yet, it seems that Israeli legislation neglects to do so. Although the Ministry of Health has established the formalized regulations analyzed in this study, we argue that the legislature should address the contradictions between these policies and existing laws that lack specificity regarding the role of family members in caring for elderly and ill relatives.

To address this gap, future policymakers could consider amending existing legislation or introducing a dedicated chapter that outlines the obligations and expectations of family members in caregiving roles within the PRA [19], the Terminally Ill Patient Law of 2005 [24], or the Guardianship Law [25]. Such amendments would not only enhance the connection and quality of care for geriatric patients but also ensure that the responsibilities of children towards aging parents are clearly defined and understood.

Implementing these changes presents several challenges, particularly given the complex dynamics of family relationships. For many families, the relationship between parents and children may be strained or, in some cases, nonexistent. Enforcing obligations in such circumstances is fraught with difficulties. Therefore, policymakers must anticipate various scenarios and develop nuanced mechanisms that can assess each case based on its context and merits. This approach would require implementing a framework that considers factors such as the relationship between parents and children, the capacity of the children to provide support, be it financial, emotional, or physical, and their willingness to engage. For instance, establishing an assessment protocol could involve consultations with social workers or mediators trained in family dynamics, helping to ensure that all parties’ needs are considered. Moreover, the proposed legislation could include provisions for support systems that empower children to fulfill their responsibilities and provide necessary resources for families. This might include training for family members, access to counseling services, or financial incentives for those who take on caregiving roles. For instance, key institutions, such as the ethics committees in medical facilities, the guardianship office in the Ministry of Justice, family courts, and social services departments, can manage and oversee such mechanisms. These bodies can intervene under circumstances such as concerns regarding the welfare of the elderly person, the individual lacking the capacity to make independent decisions, potential risks to the person’s health or safety, and family members being unable or unwilling to provide necessary care. Therefore, our call for reform in the existing legal framework is an invitation for policymakers and healthcare professionals to reevaluate existing practices and adapt them to the realities of geriatric patient care. Prioritizing family involvement in geriatric hospitalization can create a more supportive healthcare environment that acknowledges the critical role of families in the healing process and ensures that patients receive care that is aligned with their wishes and needs.

## Strengths and limitations

Understanding the interactions between family involvement, autonomy, and regulatory frameworks offers valuable insights into how institutions can better facilitate family engagement through policies and practices that foster collaboration. By addressing this gap, we hope to pave the way for a more comprehensive approach to geriatric care that prioritizes both family roles and the well-being of older adults.

However, the findings reported here do not clarify whether and to what extent family participation is adequately upheld and implemented in geriatric institutions. While policies clearly advocate for family involvement in the care process, the reality of how these policies are enacted in practice remains unclear. The gap in knowledge and research around family participation in geriatric hospitals highlights several key areas for future inquiry. First, examining the consistency of policy application across various geriatric institutions is essential. Are these policies implemented uniformly, or do they vary significantly from one institution to another? For example, future research can examine how family involvement policies are interpreted and implemented across different types of hospitals, such as urban and rural settings, central or peripheral areas, and large or small hospitals. Understanding these variations can provide insights into how organizational behavior, leadership, and various barriers influence family participation.

Resource constraints within hospitals, such as limited staff, time, and financial resources, can hinder healthcare providers’ ability to engage families adequately. Qualitative interviews with healthcare providers, geriatric patients, and family members can uncover their attitudes and perceived challenges regarding family participation and illuminate barriers that need addressing.

Staff attitudes also play a critical role in facilitating or obstructing family participation. Investigating how healthcare professionals perceive family involvement and the training they receive on this topic could offer valuable insights into promoting a more inclusive approach to geriatric care. Additionally, addressing potential conflicts that arise between patients and families, or within families themselves, is an important consideration. Understanding these dynamics can help develop strategies to mitigate conflicts and foster a supportive environment for geriatric patients.

Finally, conducting research not only in Israel but also across different societies can lead to a nuanced understanding of family involvement in geriatric care. Diverse cultural perspectives on family roles and responsibilities may impact how participation in geriatric care is perceived and practiced. Therefore, future research should explore how these values are applied in hospital settings in Israel and other societies, providing a clearer picture of family involvement in practice.

## Conclusion

The themes identified in this study reveal that the active participation of families in geriatric hospitals is not merely beneficial but essential. By prioritizing information sharing and communication, care planning and decision making, advocacy, and emotional and social support, healthcare providers can create an environment that enhances patient care while also supporting families in their critical roles. Moving forward, healthcare institutions and policy makers must develop strategies to further integrate families into the care process, ensuring that the diverse needs of geriatric patients are met holistically. Through this comprehensive approach, we aim to inspire further research and advocacy for policies that prioritize family participation in geriatric care, ultimately improving the health outcomes and quality of life of elderly individuals.

## Data Availability

No datasets were generated or analysed during the current study.

## Footnotes

1 The word “family” appeared between one and nearly twenty times per document across 84 documents. Other high-frequency codes related to family involvement included “involvement” (15 times), “children” (14 times), “relatives” (18 times), “family support” (4 times), “family involvement” (2 times), and “family participation” (3 times).
